# An Optimal Centralized Carbon Dioxide Repository for Florida, USA

**DOI:** 10.3390/ijerph8040955

**Published:** 2011-03-31

**Authors:** Brandon Poiencot, Christopher Brown

**Affiliations:** School of Engineering, University of North Florida, Building 50, 1 UNF Drive, Jacksonville, FL 32224, USA; E-Mail: Brandon.poiencot@unf.edu

**Keywords:** CCS, carbon sequestration, centralized repository, transportation optimization, Florida, storage alternatives

## Abstract

For over a decade, the United States Department of Energy, and engineers, geologists, and scientists from all over the world have investigated the potential for reducing atmospheric carbon emissions through carbon sequestration. Numerous reports exist analyzing the potential for sequestering carbon dioxide at various sites around the globe, but none have identified the potential for a statewide system in Florida, USA. In 2005, 83% of Florida’s electrical energy was produced by natural gas, coal, or oil (e.g., fossil fuels), from power plants spread across the state. In addition, only limited research has been completed on evaluating optimal pipeline transportation networks to centralized carbon dioxide repositories. This paper describes the feasibility and preliminary locations for an optimal centralized Florida-wide carbon sequestration repository. Linear programming optimization modeling is used to plan and route an idealized pipeline network to existing Florida power plants. Further analysis of the subsurface geology in these general locations will provide insight into the suitability of the subsurface conditions and the available capacity for carbon sequestration at selected possible repository sites. The identification of the most favorable site(s) is also presented.

## 1. Introduction

The potential for global climate change due to warming of the planet has been studied and debated over the last decade. The majority opinion is that the climate is changing due to emissions of carbon dioxide and other greenhouse gases. Scientists from around the globe generally agree that reducing greenhouse gas emissions is of paramount importance. One way to achieve sizable reductions over the next few decades is to deploy carbon capture and sequestration (CCS) in a mass scale [1,2]. The United States Department of Energy, and engineers, geologists, and scientists the world over have investigated the potential for reducing atmospheric carbon dioxide emissions through underground storage or geologic carbon sequestration. Although considerable research has been completed, no one has studied the potential for an integrated CCS system in Florida, USA. Florida is heavily dependent upon fossil fuels for its primary power generation. According to 2005 Environmental Protection Agency (EPA) data, there are 136 large and small power plants in Florida including those that use coal, oil, natural gas, and biomass as their source of fuel. Due to the large number of fossil fuel power plants in Florida, Florida also generates a significant mass of carbon dioxide emissions each year. In 2005, those emissions totaled almost 134,000,000 tonnes or 134 Mt [3]. In the future, new regulations are likely to require carbon dioxide emission reductions across the United States and in Florida. Florida utility companies are currently investigating feasible alternatives to reduce overall carbon dioxide emissions but most alternatives will be very expensive and difficult to implement over the short-term.

While each alternative has advantages and disadvantages, forward thinking companies are likely to consider CCS due to its potential to sequester or store massive amounts of carbon dioxide and other greenhouse gases and its relatively low-cost attributes [4]. So what is CCS and how will it help reduce carbon liability? CCS is a technological innovation whereby carbon dioxide off-gas is captured, separated from other gases, concentrated, compressed, and then injected into underground repositories. Here the carbon dioxide is sequestered or stored for hundreds to thousands of years, effectively reducing the carbon footprint of the industrial emitter. In 2005 83% of Florida’s electrical energy was produced by fossil fuels while in 2010 the percentage was almost 89% [5]. The continuing use of fossil fuels may depend upon finding suitable subsurface sequestration repositories in Florida and connecting them to a optimized network of pipelines and primary CO_2_ sources.

According to the Intergovernmental Panel on Climate Change (IPCC), storage of CO_2_ in geologic formations includes four primary storage repository categories: saline aquifers, existing oil fields, depleted natural gas fields, and thin-nonmineable coal seams [6]. The capacity of each of these repository categories to sequester CO_2_ is an important planning variable to be considered during feasibility-level investigations of potential projects [1,7]. Deep saline aquifers appear to offer the highest potential capacity of the four primary options [8–11]. In addition, in Florida, saline aquifers are the most likely storage option. According to the United States Department of Energy [12], the estimated capacity of oil/gas fields is relatively small by comparison (e.g., 100 times less) and their geographic distribution is rather limited. A typical CCS saline aquifer storage project will undergo several operational changes over time with the injected CO_2_ ultimately becoming completely dissolved in the aquifer fluid. The various operation phases include site characterization, initial active injection, post-injection, and long-term monitoring. During the project lifecycle, there are significant changes in the state of injected CO_2_ with it starting as a free-phase, becoming residually-trapped, being dissolved, and ultimately being precipitated as a mineral. The relative time scales for each process are different with residual trapping likely a decadal time scale, dissolution over hundreds of years, or more likely in saline waters, thousands of years and mineralization over even longer periods. During active operations, when liquid or supercritical CO_2_ is being injected into a repository, the CO_2_ will be highly mobile as a pure separate phase and concentrated aqueous phase [13]. CO_2_ is a highly compressible fluid compared to water and its density radically increases from 300 to 800 kg/m^3^ at pressure ranging from 10 to 25 MPa [14]. Since liquid or supercritical CO_2_ has a density less than the typical density of the saline repository fluid [15], it will be buoyant, tending to rise within the formation [16] until it intercepts a competent confining unit where it may spread laterally until it becomes trapped [17]. In some cases, depending upon formation dip, the supercritical CO_2_ may migrate updip along the confining unit.

This paper describes ongoing research being conducted at the University of North Florida in Jacksonville, Florida, which is evaluating the feasibility of CCS projects within the state. The preliminary research results of this effort are presented in this paper and include an evaluation of an optimal Florida-wide pipeline transportation network connecting the 40 largest sources of CO_2_ emissions with 5 alternate CO_2_ repositories or disposal areas. The 5 alternate repositories include a variety of saline aquifer storage zones located throughout Florida, including one offshore site located beneath the Gulf of Mexico, generally selected due to their favorable geologic properties. The optimized pipeline network is devised using “minimum cost transportation network model” methodology [18]. The use of this type of model for CO_2_ transmission in pipelines is relatively new and has not been investigated in Florida. Therefore, the first time application of the model methodology for this project in Florida is unique and provides a sound basis for more detailed evaluations in the future.

## 2. Experimental Section

### 2.1. Florida Primary Emission Sources

**Figure 1.**
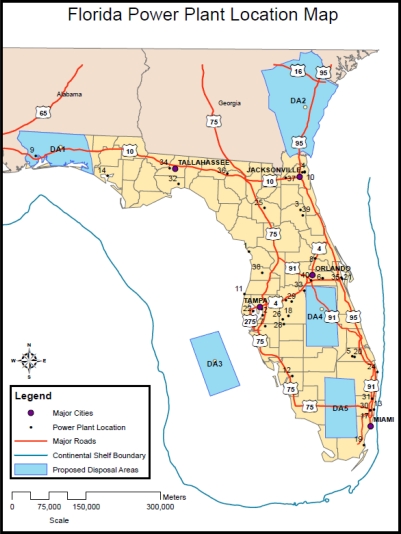
Location of 40 largest CO_2_ sources and 5 potential CCS repositories in Florida.

The first task in developing an optimal CO_2_ pipeline transportation network for Florida, is to identify the location and magnitude of the largest sources of CO_2_ within the state. Florida has approximately 136 primary sources of CO_2_ inventoried by the EPA. For the initial model development effort, the 40 largest sources of CO_2_ were identified and summarized [3]. These 40 sources comprise over 90% of the 2005 total CO_2_ emissions for Florida. The 40 sources along with a map identification number, location in UTM 1983 (meters) horizontal grid coordinates, and the respective annual CO_2_ emissions are listed in Table 1. Each of the 40 sources is also shown on Figure 1 along with 5 potential CO_2_ repositories discussed later in this paper.

### 2.2. Florida Pipeline Transportation Model

Following the identification of the major emission sources and magnitudes, the pipeline network model must be developed. The first step in this effort is to develop the pipeline cost model. Multiple investigators have developed and presented CO_2_ pipeline transportation models. Heddle *et al.* developed the initial MIT CO_2_ transport model [19]. Their model was a simple linear model that includes initial capital cost and annual operation and maintenance (O&M) costs. Equation (1) presents their model followed by an explanation of the variables.

(1) [Total Annual Cost = α×D×L×CF+O&M Cost]

where α is a constant estimated by MIT from literature values to be $33,853 (2003 dollars);

D is the pipeline diameter in inches and is function of flow rate;
L is the least-cost pipeline route length in miles; and,
CF is a capital cost factor used to annualize the initial capital cost.

McCoy developed a more sophisticated model that provides for regional cost differences as well as further resolution of cost factors such as pipe materials, labor, real estate, permitting, design, and construction management [20]. McCoy developed the cost model using regression analysis of natural gas pipeline construction projects as published in the Oil and Gas Journal between 1994 and 2003. The McCoy model is presented in Equation (2) herein.

(2) $$\left[\text{Total}\;\text{Annual}\;\text{Cost}=(\beta_{m}\times L^{a6m}\times D^{a7m}\times \text{CF})+(\beta_{L}\times L^{a6L}\times D^{a7L}\times \text{CF})+(\beta_{\text{RE}}\times L^{a6\text{RE}}\times D^{a7\text{RE}}\times \text{CF})+(\beta_{\text{MS}}\times L^{a6\text{MS}}\times D^{a7\text{MS}}\times \text{CF})+O\&M\;\text{Cost}\right]$$

where β_m_, β_L_, β_RE_ and β_MS_ are cost coefficients for materials, labor, real estate, and miscellaneous (e.g., design, permitting, construction management) in 2004 dollars; L is the least-cost pipeline route length in kilometers; D is the pipeline diameter in inches and is a function of flow rate, inlet pressure, outlet pressure, and frictional losses; CF is a capital cost factor used to annualize the initial capital cost; a6_m_, a6_L_, a6_RE_, and a6_MS_ are model pipeline length power exponents for materials, labor, real estate, and miscellaneous (discussed further below); and, a7_m_, a7_L_, a7_RE_, and a7_MS_ are model pipeline diameter power exponents for materials, labor, real estate, and miscellaneous (discussed further below).

In addition to MIT and McCoy, several others have developed recent cost models including Skovholt, Bakken & Von Streng Velken, and Zhang *et al.* [21–23]. Other recent papers have focused upon more sophisticated CCS infrastructure models [24–28]. These models simulate the full CCS value chain including carbon capture, compression, transport, and storage. For example, Mendelevitch *et al.* (2010) includes the “full decision path” for CCS infrastructure decisions in addition to consideration of a carbon tax or CO_2_ credits [24]. Kuby *et al.* (2011) discusses a CCS simulator that “determines the optimal quantity of CO_2_ to capture and optimize the various components of a CCS infrastructure network, given the price per tonne to emit CO_2_ into the atmosphere” [29]. All of the models are similar with capital costs dependent upon pipeline diameter and length. A key part of these models is the methodology to estimate the pipeline diameter. For most of these models, pipeline mass flow rate and diameter is a function of inlet pressure, outlet pressure, frictional losses, and topography (or elevation change). Since Florida is relatively flat, topographic differences are expected to be minor. In addition, if general transportation pressures are assumed to be consistent with industry practice, the development of a generalized method of estimating the required pipe diameter for a given mass flow rate is feasible. With the exception of Bakken & Von Streng Velken, the models that have been discussed in this paper so far have focused on landbased pipeline networks [22].

**Figure 2.**
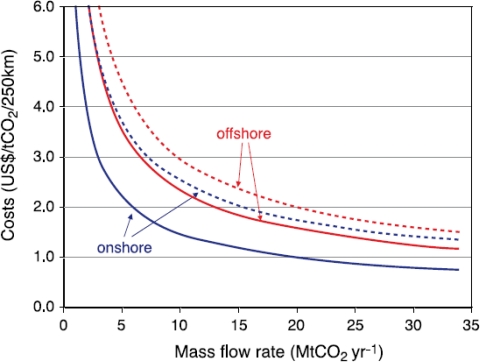
CO_2_ Pipeline transportation costs per tonne for 250 kilometer pipeline *versus* mass flow rate in Mt CO_2_ per year (After IPCC (2005, Chapter 4, Figure 4.5)).

The IPCC presents pipeline transport costs over land and underwater *versus* CO_2_ mass flow rate [6]. This figure is reproduced herein for discussion purposes as Figure 2. As shown on the figure the cost relationship is highly nonlinear and the cost envelop is not constant. At a mass flow rate of 5 Mt per year, the cost envelope ranges from a lower bound of $2.10 for land construction and an upper bound of $4.50 for underwater construction. In addition, the costs for underwater pipeline are considerably more expensive per kilometer than land construction. In comparing the lower bound for land construction with the lower bound cost for underwater construction, underwater construction is 50 to 75% more expensive. Bakken & Von Streng Velken present a cost model for a CCS project in Norway that is completely planned to be underwater [22]. In comparing these unit costs to the MIT model or McCoy model costs above, the unit cost is more than 2.5 times greater than equivalent land pipeline construction. In order to develop a new cost model for Florida, the percentages of land and underwater pipeline need to be calculated and a cost differential applied. This cost factor was assumed to be 1.75 and is discussed further below.

**Figure 3.**
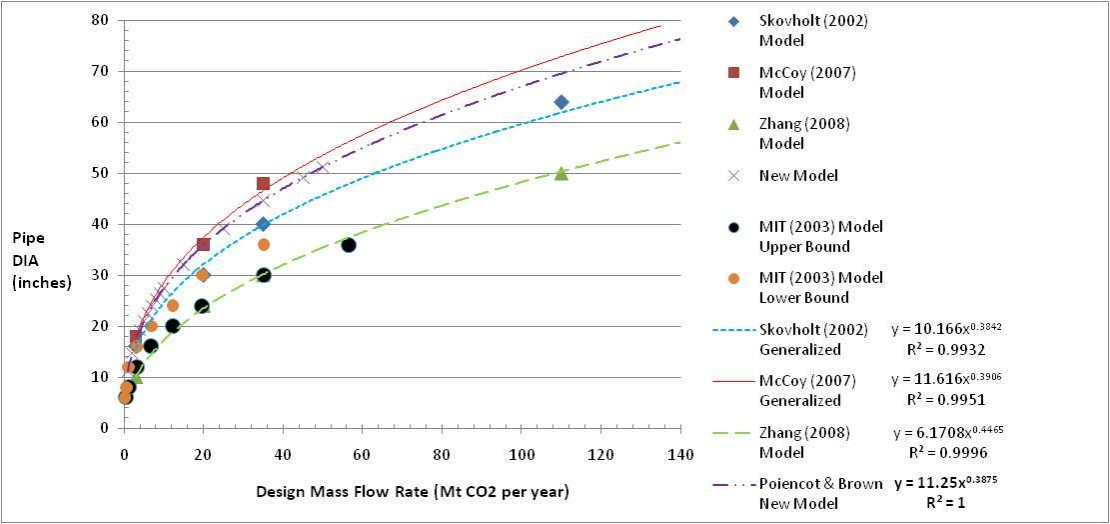
CO_2_ Pipeline Diameter (inches) *versus* CO_2_ Mass Flow Rate Megatonne (Mt) CO_2_ per year for Multiple Published Cost Models (DIA = Diameter on Axis label).

For this paper, as part of pipeline cost model development, the authors noted that all of the cost models require considerable calculations in order to estimate the required pipeline diameter. These calculations are certainly required for detailed pipeline design efforts but simpler estimates probably will suffice for planning or feasibility studies. The authors plotted pipeline diameter *versus* mass flow rate for several of the models discussed in order to develop a more generalized feasibility-level approach for estimating the pipeline diameter. Figure 3 presents the results of the various models. The authors fit published data to similar power models to ascertain a reasonable generalized model for the current research effort. The curve fitting statistics or R^2^ coefficients for the various power models were all greater than 0.99. For this research effort the authors chose to use a model between Skovholt and McCoy [20,21]. It is also interesting to note that the Zhang *et al.* model returns practically the same values as the Heddle *et al.*/MIT “upper bound” diameter model [19,23]. The new proposed model provides a simplistic way to estimate the necessary pipeline diameter given solely an estimate of the required CO_2_ mass flow rate. The new model was developed for both English and SI units to permit practitioners from around the globe to use the model for preliminary planning purposes. Equations (3) and (4) provide the diameter estimation models in English and SI units, respectively.

(3) $$\left[\text{Pipeline}\;\text{Diameter},D=(11.25\times {ɛ}^{0.3875})\right]$$

where ɛ is the CO_2_ mass flow rate in Megatonnes (Mt) per year; D is the pipeline diameter in inches.

(4) $$\left[\text{Pipeline}\;\text{Diameter},D=(0.2857\times {ɛ}^{0.3875})\right]$$

where ɛ is the CO_2_ mass flow rate in Mt per year; D is the pipeline diameter in meters.

Other considerations for a new pipeline cost model are also important. First, the previous models were all developed in different years so that inflation adjustments are necessary to bring all models to 2010 costs. Lewis provides construction cost factor data from 1996 to March 2010 for skilled labor, common labor, and materials [30]. From April 2004 to March 2010, the construction cost adjustment factors are 1.18 for materials, 1.25 for common labor, and 1.26 for skilled labor (e.g., for designers, permit specialists and construction managers). Real Estate costs have receded to close to 2005 costs such that a cost adjustment factor of only 1.05 was assumed for the new model. In addition, pipelines may ultimately be required on land and underwater so a cost differential factor must be applied for underwater pipelines. As noted previously, the value of 1.75 was adopted for this study to convert a pipeline cost on land to one underwater. This value represents a reasonable average factor as determined from the literature. Lastly, the actual pipeline O&M cost needs to be determined. For detailed design, precise estimates of energy requirements, system maintenance, and inspection are required. For preliminary modeling purposes it is acceptable to use mean cost values from the literature as was done for this research effort. Using normalized unit O&M costs in $/tonne CO_2_/kilometer from Zhang *et al.* and Bakken & Von Streng Velken, the authors developed a reasonable mean O&M cost of 0.0088 $/tonne CO_2_/kilometer [22,23]. In reviewing the IPCC costs shown in Figure 2 above, one can calculate a similar mean O&M cost for comparison purposes [6]. Normalized O&M costs range from 0.005 $/tonne CO_2_/km for 30 Mt per year to 0.014 $/tonne CO_2_/km for 5 Mt per year. Mendelevitch *et al.* (2010) uses a value of 0.014 $/tonne CO_2_/km [24]. Therefore, the adopted value of 0.0088 $/tonne CO_2_/km seems reasonable for a feasibility-level model especially since it is based upon more recent data than the original IPCC figure. With all of the required cost model elements assembled, the authors chose to adapt the McCoy model for use in Florida since it was thought to be the most complete and flexible [20]. Equation (5) provides the proposed new Florida CO_2_ pipeline cost model.

(5) $$\begin{array}{c} {}[\text{Total}\;\text{Annual}\;\text{Cost}=[[\omega_{\text{m}}\times \beta_{\text{m}}\times {\text{L}}^{\text{a}6\text{m}}\times {\left(\text{D}\times 39.38\right)}^{\text{a}7\text{m}}\times \text{CF}]+\\ \left[\omega_{\text{L}}\times \beta_{\text{L}}\times {\text{L}}^{\text{a}6\text{L}}\times {\left(\text{D}\times 39.38\right)}^{\text{a}7\text{L}}\times \text{CF}]+[\omega_{\text{RE}}\times \beta_{\text{RE}}\times {\text{L}}^{\text{a}6\text{RE}}\times {\left(\text{D}\times 39.38\right)}^{\text{a}7\text{RE}}\times \text{CF}\right]+\left[\omega_{\text{MS}}\times \beta_{\text{MS}}\times {\text{L}}^{\text{a}6\text{MS}}\times {\left(\text{D}\times 39.38\right)}^{\text{a}7\text{MS}}\times \text{CF}\right]+\\ \left[0.0088\times ɛ2\times \text{L}]]\times \alpha \right] \end{array}$$

where ω_m_, ω_L_, ω_RE_ and ω_MS_ are cost adjustment coefficients to convert April 2004 costs to March 2010 costs and are ω_m_ = 1.18, ω_L_ = 1.15, ω_RE_ = 1.05, and ω_MS_ = 1.26; β_m_, β_L_, β_RE_, and β_MS_ are cost coefficients for materials, labor, real estate, and miscellaneous (e.g., design, permitting, construction management) in 2004 dollars and are β_m_ = 1,534.62, β_L_ = 30,690.22, β_RE_ = 8,912.51, and β_MS_ = 33,265.96; L is the least-cost pipeline route length in kilometers; D is the pipeline diameter in meters and is a function of flow rate (see Equation (4) above); CF is a capital cost factor of 0.067574 assuming a 5% discount rate used to annualize the initial pipeline capital construction cost; ɛ2 is CO_2_ mass flow rate in tonnes per year; α is a factor to adjust costs for underwater construction, it is 1.75 for underwater projects and 1.0 for land pipeline projects; a6_m_, a6_L_, a6_RE_, and a6_MS_ are model pipeline length power exponents for materials, labor, real estate, and miscellaneous and are a6_m_ = 0.901, a6_L_ = 0.82, a6_RE_ = 1.049, and a6_MS_ = 0.783; and, a7_m_, a7_L_, a7_RE_, and a7_MS_ are model pipeline diameter power exponents for materials, labor, real estate, and miscellaneous and are a7_m_ = 1.59, a7_L_ = 0.94, a7_RE_ = 0.403, and a7_MS_ = 0.791. The new cost model for Florida is intended for use as a planning tool to be used in feasibility-level studies. It is applicable for use in Florida or other areas of similar flat topography.

### 2.3. Geologic Repository or Disposal Zones

With the pipeline cost model developed and the sources or supply nodes identified, the CCS repository or demand locations are identified next. The location was based upon the available geology, location of existing emission sources, and institutional concerns regarding possible CO_2_ releases [31]. Based upon the existing research, Florida has ample potential CCS repositories including depleted oil/gas fields, unminable coal seams, and deep, saline aquifers [32–38]. Of the four primary disposal alternatives, saline aquifers present the best opportunity to store large quantities of CO_2_ safely [4,12]. Building upon the existing research, this paper has chosen 5 separate saline aquifer CCS repository sites (see Figure 1) distributed throughout Florida and Southeast Georgia. Each of the 5 sites represents a portion of an identified CO_2_ disposal/repository site outlined in the “2010 Carbon Sequestration Atlas of the United States and Canada” [12]. The actual capacity of each of these prospective areas needs to be confirmed through further numerical modeling and subsurface testing [39,40]. Capacities assumed for the optimization modeling in this paper are on the low end of regional estimates. Each of these 5 sites is discussed herein.

The Florida panhandle contains ample potential capacity for carbon sequestration within the Upper Cretaceous Zone, specifically the Tuscaloosa Formation. This formation is present in several Gulf Coast states and is estimated to have a “low” estimate capacity of at least 5 Gt according to the [12]. For this study, the capacity within Florida (DA1 on Figure 1) was estimated by the area-weighted share of the total estimated low capacity or 215 Mt. The top of the Tuscaloosa Formation is usually found below the shales of the overlying Eutaw Formation and in the area of Cedar Keys, Florida consists dominantly of red, light red, brown or mottled shales with interbedded sandstones [32,38]. Raymond and Copeland state in the Coastal Plain Province of Alabama, the Tuscaloosa Group comprises mainly fossilferous, nearshore, marine clastics [37]. In eastern-most Alabama, the formation is typically poorly sorted kaolinitic, arkosic sand and gravel interbedded yellowish-orange to reddish-green mottled kaolinitc clay. Thickness of the Tuscaloosa Formation ranges anywhere from 100 to 400 meters [37]. USDOE describes the proposed storage reservoir at Southern Company Plant Daniel in Mississippi as a “massive sandstone that is a thick, regionally extensive, porous and permeable coastal to deltaic-marine sandstone at the base of the lower Tuscaloosa” [12]. According to the report, the lower Tuscaloosa in this area is overlain by a thick section of 90 to 140 meters of shales and mudrocks that were deposited as sea level rose during a marine transgression.

DA2, shown on Figure 1, covers southeast Georgia. The proposed repository, the South Carolina-Georgia Basins, is located in saline aquifers that exist in the upper cretaceous layers of southeast Georgia, including the Atkinson Formation. The total preliminary “low” estimate capacity of this repository zone is 12.6 Gt [12]. For this study, the capacity (DA2 on Figure 1) was estimated by the area-weighted share of the total estimated low capacity or 4.98 Gt. The Atkinson formation is typically split into two separate units [34]. The separation of the units occurs roughly 990 meters from the surface [34]. The top unit tends to consist of shale which is micaceous, glauconitic and phosphoritic with the occasional non-uniform grain size sandstones. The lower unit of the Atkinson Formation, or the Basal Sand Unit, is a quartz sandstone, fine to coarse grained, poorly sorted, soft and typically glauconitic, phosphoritic and micaceous [34]. The Basal Sand Unit does contain sporadic stringers of shale, silt, and limestone. It is assumed that CO_2_ disposal would primarily focus on the Basal Sand Unit.

The repositories located in the peninsular region of Florida (DA4) and the offshore location (DA3) would inject CO_2_ into the Cedar Keys/Lawson Dolomite formations. USDOE estimates that the entire Cedar Keys/Lawson Dolomite formations capable of storing CO_2_ have a “low” estimate capacity of approximately 11 Gt [12]. For this study, the capacity of each area (DA3 and DA4 on Figure 1) was estimated by the area-weighted share of the total estimated low capacity or 1 Gt. According to Chen, the Cedar Keys Formation is widely spread across peninsular Florida and spreads into the panhandle [33]. In Brevard County, Florida, the top of the Cedar Keys Formation ranges from approximately 670 meters NGVD to 914 meters NGVD below land surface. The formation consists of dolomite and evaporates with a minor amount of limestone. Gypsum commonly fills pore spaces within the dolomite beds and occurs as thin irregular streaks or seams in the dolomite [33]. The Lawson Formation is generally found at the base of the Cedar Keys Formation. The Lawson is comprised mainly of pure, clean, very light brown and fine crystalline dolomite and/or chalky dolomitic limestone [33].

The disposal area indicated in south central Florida (DA5) has the potential to utilize multiple “stacked” secondary formations as a repository. USDOE estimates that the entire Cedar Keys/Lawson Dolomite formations capable of storing CO_2_ have a “low” capacity of approximately 11 Gt [12]. For this study, the capacity of the area (DA5 on Figure 1) was estimated by the area-weighted share of the total estimated low capacity or 1 Gt. Including other stacked zones in the Paleocene and Eocene would contribute additional capacity for storing CO_2_, however, in order to provide a conservative estimate, this capacity was not included in the capacity estimate. In south Florida, zones of high transmissivity exist within the dolomite units of the Eocene, Paleocene, and Upper Cretaceous Formations [36]. The high transmissivity of the region is attributed to small to large cavities and caverns within the dolostone formations [35]. The cavities and caverns in the Upper Cretaceous Formation are typically found near the base of a massive dolostone facies in the upper part of the system and range in size from a baseball to a small car [36]. Data from nearby wells indicate the host rock is light to dark brown, anhedral dolestone. The same lithology holds true for the Paleocene and Eocene Cavity Zones [36]. The caverns tend to be surrounded by nearly impermeable material [35]. Utility companies local to the area have used the “Boulder Zone” for wastewater disposal in the past. Again, for the purpose of this study, these zones were not included in the storage estimates for area DA5.

### 2.4. Optimization Model

After the supply nodes and the demand nodes are identified and characterized, the transportation network is developed connecting supply nodes with demand nodes. Essentially, the least-cost transportation model can be developed using linear programming whereby a set of supply sources is linked to a set of demand locations with an optimal pipeline route where the unit costs of transportation between supply-demand pairs is known [18]. The primary model includes 40 possible CO_2_ supply sources each of which can be transported to any of 5 potential CCS demand locations or CCS repositories. Therefore, for the preliminary model there are 200 unknown CO_2_ “flows” for each year that must be solved using a set of linear equations. Each supply source can supply less than or equal to its annual CO_2_ emissions to the pipeline network. Each demand location can only accept less than or equal to its designated CCS capacity. The Florida pipeline transportation cost model is an adaptation of a logistics warehousing-type model developed by Cormier and Gunn and a CO_2_ pipeline optimization cost model developed by Bakken & Von Streng Velken [22,41]. This model is discussed below. The basic model equation and model constraints are included herein:

(6) $$\left[\text{Minimize}\;\sum F_{\text{ijk}}\times X_{\text{SiDjk}}=\text{Total}\;\text{Cost}\right]$$

where X is the annual CO_2_ pipeline transportation cost ($/tonne CO_2_) from CO_2_ supply node S_i_ (from i = 1 to 40) to demand node or repository D_j_ (from j = 1 to 5) at Time Year k (from k = 1 to 25 years) and F_ijk_ is the CO_2_ flow through that pathway in tonnes CO_2_/year during Year k.

(7) $$[\text{Subject}\;\text{to}\;\text{Constraint}\;1\sum_{1}^{200}\text{Fijk}\leq \text{Capacity}\;D_{j}]\;\text{Summed}\;\text{from}\;1:200\;\text{each}\;\text{year}$$

(8) $$[\text{Subject}\;\text{to}\;\text{Constraint}\;2\sum_{1}^{200}\text{Fijk}\leq \text{Emission}\;\text{Supply}\;\text{from}\;S_{i}]$$

(9) $$[\text{Subject}\;\text{to}\;\text{Constraint}\;3\sum_{1}^{200}\text{Fijk}\geq 0]$$

Using the new Florida cost model above, 200 annual CO_2_ pipeline unit transportation costs (X) were developed as part of the model development effort. These costs are shown on Table 2.

**Figure 4.**
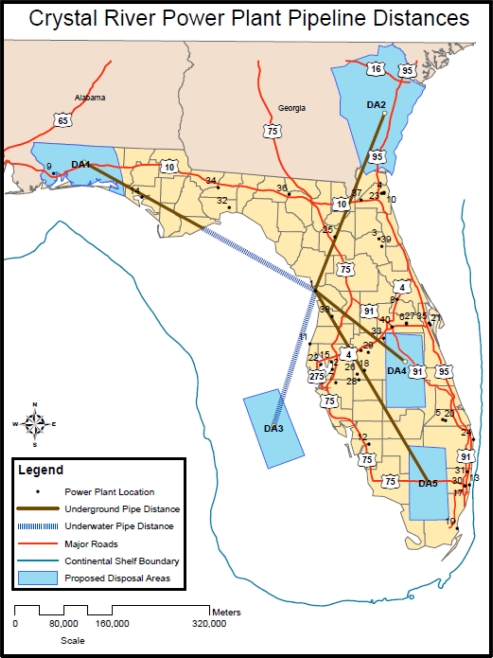
Least-cost pipeline transportation network example.

Each of the unit costs was used to simulate the least-cost transportation network. In this first modeling effort, the least-cost transportation network was not constrained by geography, real estate limitations, institutional concerns (e.g. location of wetlands, parks, sensitive natural areas), or practical engineering considerations regarding pipeline right-of-way selection. Each possible emission source (S_i_) was connected to each possible disposal repository (D_j_) creating five possible pathways from each emission source. The optimization model calculated the least-cost alternative for each source-disposal pair. An example of this logic is shown on Figure 4 using the Crystal River plant as an illustrative emission source. Each pipeline route is broken into landward and underwater segments to demonstrate the importance of each part in the overall cost structure with underwater pipelines costing almost twice that of landward pipelines. The unit costs for each route range from $0.99 to 36.23 per tonne CO_2_ with the arithmetic mean value at $10.49 per tonne per CO_2_. For the least-cost model as determined through the optimization effort, the total levelized cost for the network ranged from $2.29 (during Year 1 for example) to $3.99 per tonne CO_2_ (during Year 25) which is in the range of $1 to $7 per tonne CO_2_ referenced by McCoy [20]. As repositories are filled up beyond 25 years, the levelized cost could rise as high as $6.18 per tonne CO_2_.

## 3. Results and Discussion

**Figure 5.**
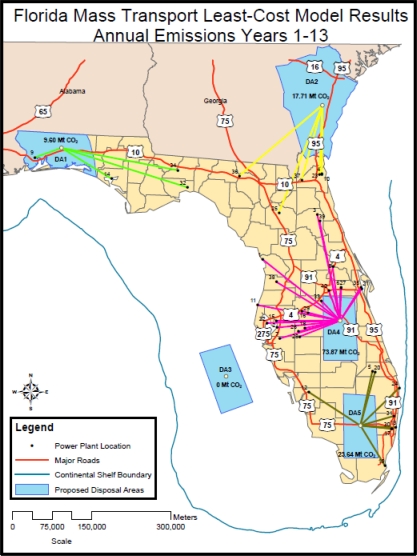
Simulation results Years 1 to 13.

Following model development and testing, the preliminary optimal pipeline network was determined. Due to the high CO_2_ disposal area capacity at each proposed Florida repository location, the least-cost network model resulted in the use of all 5 repositories. Initially, in years 1 to 13, the proposed offshore repository was not utilized at all in the optimal network due to high unit costs for underwater pipeline installations. The underwater cost differential would have to be reduced by more than 30% for the offshore repository to be cost-effective during this timeframe. However, at the beginning of year 15, DA4 capacity is filled and CO_2_ flows initially directed to DA4 shift to DA2, DA3, and DA5. During years 1 to 13, the model indicated that approximately 7.62% of the modeled emissions were transported to DA1, 14.05% to DA2, 58.63% to DA4, and 19.70% to DA5. The results for years 1 to 13 are shown in Table 3 and graphically presented on Figure 5. In year 14, as DA4 fills up, the model indicated that approximately 7.62% of the modeled emissions were transported to DA1, 34.09% to DA2, 7.28% to DA3, 31.32% to DA4, and 19.70% to DA5. In years 15 to 22, the model indicated that approximately 7.62% of the modeled emissions were transported to DA1, 38.39% to DA2, 16.45% to DA3, and 37.54% to DA5. During year 23, DA1 is filled to capacity. In year 23, as DA1 fills up, the model indicated that approximately 3.04% of the modeled emissions were transported to DA1, 42.97% to DA2, 16.45% to DA3, and 37.54% to DA5. During the last 2 years of the model simulation, only three repositories are available and the remaining flows that went to DA1 instead are directed to DA2. During the initial years of the simulation (years 1 to 13) none of the 5 initial disposal areas is filled within the simulation period, repository capacity was not a model constraint so each of the 4 sites that were utilized received CO_2_ from emission sources closest to that site. For example, DA2 received all of its CO_2_ from large power plants in the Jacksonville, Florida area. Likewise, DA5 received all of its CO_2_ from large power plants in the Miami-Fort Lauderdale, Florida area. Additional research is warranted regarding maximum injection rates that can be sustained in each repository area as this may also affect the ultimate site feasibility. The optimal network included 5 out of the 5 simulated repository sites. If only one of the sites could be permitted and developed [42], it appears the most favorable site within Florida would be DA4, located south of Interstate 4 in Osceola County, Florida. This repository site will be further explored in future modeling to be completed as part of this research effort since under current capacity estimates; this site is filled within 14 years. One additional modeling scenario was generated that assumed that repositories 1, 4, and 5 were either filled or could not be permitted. Under these assumptions, almost 65% of the CO_2_ emissions would be transported to DA2 with the remainder going to DA3. Since a large flow is targeted to DA3, the offshore site, unit levelized costs would almost triple to $6.18 per tonne of CO_2_. In addition, future modeling efforts will evaluate an alternate pipeline network for Florida that will likely run along existing transportation right-of-ways (ROW) within Florida. This network will also combine smaller emission sites into “emission zones” where smaller contributing power plants will simply tap into larger diameter CO_2_ pressure mains emanating from the major emission loads. It is expected that this next generation optimization model will further reduce the overall projected cost for a Florida-wide pipeline network. Future modeling efforts may also need to include allowances for emission growth as Florida has experienced in the past. Florida’s gross GHG emissions are rising faster than those of the nation as a whole (gross emissions exclude carbon sinks, such as forests). Florida’s gross greenhouse gas emissions increased by about 35% from 1990 to 2005, while national emissions rose by 16% from 1990 to 2005 [43]. The growth in Florida’s emissions from 1990 to 2005 is primarily associated with the electricity consumption and transportation sectors [43]. The effect of alternative energy and nuclear projects under development in Florida may also impact the analysis by actually reducing the overall emissions. Future modeling efforts should also include a factor that weights the emission sources by their probability of using CCS. Also, future models should also evaluate the actual surface “footprint” of the CO_2_ in order to refine the estimated repository capacity [44].

## 4. Conclusions

In conclusion, this paper discusses the development of and preliminary results from, a model of a Florida-wide CO_2_ pipeline transportation network. The top 40 primary CO_2_ emission sources in Florida were linked to 5 hypothetical repositories spread throughout the state including one offshore site within the Gulf of Mexico. The optimization model determined that an optimal network of 5 sites results in the lowest preliminary network cost which is estimated to cost approximately $289,000,000 to $503,000,000 per year for 25 years. During years 1 to 13 when all repositories are available for disposal, the total annual levelized cost is $2.26 per tonne CO_2_. As repository capacity fills up, this levelized cost rises to $2.58 per tonne in year 14; $3.52 per tonne in years 15 to 22; $3.76 per tonne in year 23; and, $3.99 per tonne in years 24 to 25. Beyond the 25-year simulation period, it is estimated that only DA2 and DA3 would have remaining capacity to store CO_2_ and the levelized cost would increase further to $6.18 per tonne CO_2_. This preliminary modeling effort suggests that a fully-connected pipeline network would not be the lowest cost CCS plan as long as all five repository locations are available.

## Figures and Tables

**Table 1.** 40 Largest sources of CO_2_ emissions in Florida [3].

**Map ID**	**Plant/Facility Name**	**Northing (UTM)**	**Easting (UTM)**	**Annual CO_2_ Emission (Mt)**
1	Crystal River	3,204,678.619	334,313.2096	15.74
2	Big Bend	3,075,217.595	361,725.5861	9.13
3	Seminole	3,289,401.756	438,698.3555	9.10
4	St Johns River Power Park	3,366,685.189	447,107.3266	8.56
5	Martin	2,992,447.289	543,356.5439	7.82
6	Stanton Energy Center	3,150,786.762	483,497.4057	6.46
7	Manatee	3,054,259.052	367,211.8689	5.84
8	Sanford	3,190,513.316	468,238.3524	5.42
9	Crist	3,398,084.815	–97,895.92908	5.12
10	Northside Generating Station	3,365,145.618	446,936.553	4.96
11	Anclote	3,118,132.981	324,756.7577	3.91
12	Fort Myers	2,953,082.051	422,095.7715	3.79
13	Port Everglades	2,885,457.356	587,476.496	3.48
14	Lansing Smith	3,357,948.163	47,642.89122	3.25
15	H. L. Culbreath Bayside	3087854.701	360,314.9618	3.03
16	C D McIntosh Jr	3,106,510.129	409,058.5118	3.01
17	Lauderdale	2,883,472.218	580,187.5679	2.41
18	Hines Energy Complex	3,074,088.024	414,350.2864	2.30
19	Turkey Point	2,813,351.444	567,289.7214	2.25
20	Indiantown Cogeneration LP	2990837.399	548,351.095	1.99
21	Cape Canaveral	3,149,224.713	523,083.2452	1.88
22	P L Bartow	3,083,763.645	342,512.6343	1.86
23	Cedar Bay Generating Company LP	3,365,190.106	442,547.4555	1.69
24	Riviera	2,960,791.27	594,173.507	1.66
25	Deerhaven Generating Station	3,292,844.416	365,772.0839	1.59
26	Polk	3,067,530.872	402,444.7148	1.24
27	Curtis H Stanton Energy Center	3,151,285.136	483,605.7691	1.13
28	Payne Creek	3,057,882.912	405,050.8373	0.80
29	Osprey Energy Center	3,103,281.781	420,562.9754	0.73
30	Wheelabrator South Broward	2,883,489.47	579,387.226	0.72
31	Wheelabrator North Broward	2,907,795.911	583,891.3521	0.70
32	S O Purdom	3,341,056.505	191,654.8001	0.67
33	Intercession City	3,126,192.519	446,298.0256	0.56
34	Arvah B Hopkins	3,373,808.201	173,480.9335	0.56
35	Indian River	3,151,869.056	521,286.9994	0.54
36	Suwannee River	3,362,512.556	290,459.4867	0.47
37	Brandy Branch	3,354,282.623	408,799.7506	0.44
38	Central Power & Lime	3,162,005.233	360,802.8878	0.42
39	Putnam	3,277,742.491	443,310.436	40 .40
40	Orlando Cogen LP	3,145,979.526	460,067.822	0.37

**Table 2.** Model CO_2_ pipeline unit transportation costs in Florida.

**Map ID**	**Plant/Facility Name**	**Costs to DA1 ($/tonne CO2)**	**Costs to DA2 ($/tonne CO2)**	**Costs to DA3 ($/tonne CO2)**	**Costs to DA4 ($/tonne CO2)**	**Costs to DA5 ($/tonne CO2)**
1	Crystal River	$5.38	$3.94	$5.16	$2.42	$4.57
2	Big Bend	$7.19	$5.89	$3.39	$1.79	$3.48
3	Seminole	$6.83	$2.97	$6.30	$2.94	$5.62
4	St Johns River Power Park	$6.91	$1.93	$7.57	$4.00	$6.67
5	Martin	$13.34	$9.86	$5.38	$1.75	$1.63
6	Stanton Energy Center	$10.76	$6.30	$5.49	$1.06	$4.10
7	Manatee	$14.85	$6.81	$3.29	$2.00	$3.63
8	Sanford	$10.28	$4.89	$6.57	$1.77	$4.93
9	Crist	$0.99	$8.63	$14.70	$16.25	$19.12
10	Northside Generating Station	$7.79	$2.29	$8.74	$4.56	$7.55
11	Anclote	$12.62	$6.81	$4.76	$2.93	$5.24
12	Fort Myers	$17.42	$9.16	$4.90	$2.76	$2.24
13	Port Everglades	$19.49	$14.31	$8.33	$4.23	$1.37
14	Lansing Smith	$2.27	$7.64	$13.58	$12.92	$19.54
15	H. L. Culbreath Bayside	$14.00	$7.69	$4.96	$2.50	$4.90
16	C D McIntosh Jr	$14.33	$7.25	$5.56	$1.62	$4.70
17	Lauderdale	$22.36	$16.09	$9.34	$4.74	$1.42
18	Hines Energy Complex	$16.43	$8.54	$5.63	$1.66	$4.56
19	Turkey Point	$24.52	$16.94	$10.52	$6.02	$2.08
20	Indiantown Cogeneration LP	$20.18	$14.64	$8.34	$2.79	$2.55
21	Cape Canaveral	$15.85	$12.05	$8.99	$1.87	$5.87
22	P L Bartow	$17.95	$9.61	$5.21	$3.84	$6.58
23	Cedar Bay Generating Company LP	$10.92	$3.32	$12.71	$6.57	$10.74
24	Riviera	$23.41	$20.45	$10.15	$4.18	$2.66
25	Deerhaven Generating Station	$9.89	$5.40	$12.36	$5.75	$9.94
26	Polk	$21.11	$10.94	$6.93	$2.51	$5.85
27	Curtis H Stanton Energy Center	$19.39	$13.08	$10.27	$2.05	$7.35
28	Payne Creek	$26.41	$13.54	$8.46	$3.07	$6.83
29	Osprey Energy Center	$25.63	$12.75	$10.03	$2.57	$8.11
30	Wheelabrator South Broward	$36.23	$26.41	$15.58	$7.86	$2.40
31	Wheelabrator North Broward	$35.16	$27.09	$15.63	$7.35	$2.69
32	S O Purdom	$8.86	$10.44	$21.76	$16.57	$25.78
33	Intercession City	$28.46	$13.68	$12.71	$2.49	$9.63
34	Arvah B Hopkins	$8.79	$11.39	$23.75	$16.46	$28.13
35	Indian River	$27.11	$20.89	$15.79	$3.43	$10.27
36	Suwannee River	$13.92	$8.98	$25.06	$13.67	$20.38
37	Brandy Branch	$18.71	$6.90	$24.85	$11.92	$19.36
38	Central Power & Lime	$30.05	$14.89	$14.36	$6.86	$13.76
39	Putnam	$21.48	$10.22	$20.70	$9.18	$17.20
40	Orlando Cogen LP	$34.22	$17.86	$17.12	$3.56	$12.54

**Table 3.** Preliminary optimal CO_2_ pipeline network for Florida.

**Map ID**	**Plant/Facility Name**	**Annual CO_2_ Emission (Mt)**	**Optimum Disposal Area in Florida (DA 1 to 5)**
1	Crystal River	15.74	DA4
2	Big Bend	9.13	DA4
3	Seminole	9.10	DA4
4	St Johns River Power Park	8.56	DA2
5	Martin	7.82	DA5
6	Stanton Energy Center	6.46	DA4
7	Manatee	5.84	DA4
8	Sanford	5.42	DA4
9	Crist	5.12	DA1
10	Northside Generating Station	4.96	DA2
11	Anclote	3.91	DA4
12	Fort Myers	3.79	DA5
13	Port Everglades	3.48	DA5
14	Lansing Smith	3.25	DA1
15	H. L. Culbreath Bayside	3.03	DA4
16	C D McIntosh Jr	3.01	DA4
17	Lauderdale	2.41	DA5
18	Hines Energy Complex	2.30	DA4
19	Turkey Point	2.25	DA5
20	Indiantown Cogeneration LP	1.99	DA5
21	Cape Canaveral	1.88	DA4
22	P L Bartow	1.86	DA4
23	Cedar Bay Generating Company LP	1.69	DA2
24	Riviera	1.66	DA5
25	Deerhaven Generating Station	1.59	DA2
26	Polk	1.24	DA4
27	Curtis H Stanton Energy Center	1.13	DA4
28	Payne Creek	0.80	DA4
29	Osprey Energy Center	0.73	DA4
30	Wheelabrator South Broward	0.72	DA5
31	Wheelabrator North Broward	0.70	DA5
32	S O Purdom	0.67	DA1
33	Intercession City	0.56	DA4
34	Arvah B Hopkins	0.56	DA1
35	Indian River	0.54	DA4
36	Suwannee River	0.47	DA2
37	Brandy Branch	0.44	DA2
38	Central Power & Lime	0.42	DA4
39	Putnam	0.40	DA4
40	Orlando Cogen LP	0.37	DA4

## References

[1] Bradshaw J, Bachu S, Bonijoly D, Burruss R, Holloway S, Christensen NP, Mathiassen OM (2007). CO_2_ storage capacity estimation: Issues and development of standards. Int. J. Greenh. Gas Control.

[2] (2007). The Future of Coal: Options for a Carbon Constrained World.

[3] Environmental Protection Agency (2007). Year 2005 eGRID Plant, Boiler, and Generator Data Files.

[4] United States Department of Energy (DOE) (2008). Methodology for Development of Geologic Storage Estimates for Carbon Dioxide, 2008.

[5] Energy Information Administration (EIA) Florida State Energy Profile Data 2009 and 2010.

[6] Benson S, Cook P (2005). Underground Geological Storage, Special Report on Carbon Dioxide Capture and Storage.

[7] Koide HG, Tazaki Y, Noguchi Y, Nakayama S, Iijima M, Ito K (1992). Subterranean containment and long-term storage of carbon dioxide in unused aquifers and in depleted natural gas reservoirs. Energy Conv. Manage.

[8] Bachu S, Gunter WD, Perkins EH (1994). Aquifer disposal of CO_2_: Hydrodynamic and mineral trapping. Energy Conv. Manage.

[9] van der Meer LGH (1995). The CO_2_ storage efficiency of aquifers. Energy Conv. Manage.

[10] Obdam A, van der Meer LGH, May F, Kervevan C, Bech N, Wildenborg A, Gale J, Kaya Y (2003). Effective CO_2_ Storage Capacity in Aquifers, Gas Fields, Oil Fields and Coal Fields.

[11] Herzog H, Helm D, Hepburn C (2009). Carbon Dioxide Capture and Stoage. Economics and Politics of Climate Change.

[12] United States Department of Energy (DOE) (2010). Carbon Sequestration Atlas III of the United States and Canada.

[13] Bachu S, Adams JJ (2003). Sequestration of CO_2_ in geological media in response to climate change: Capacity of deep saline aquifers to sequester CO_2_ in solution. Energy Conv. Manage.

[14] Han WS, McPherson BJ (2009). Optimizing geologic CO_2_ sequestration by injection in deep saline formations below oil reservoirs. Energy Conv. Manage.

[15] Sharqawy MH, Lienhard JH, Zubair SM (2010). Thermophysical properties of seawater: A review of existing correlations and data. Desal. Water Treat.

[16] (2010). Carbon Dioxide Thermophysical Property Calculator.

[17] Flett MA, Gurton RM, Taggart IJ, Rubin ES, Keith DW, Gilboy CF, Morris T, Thambimuthu K (2005). Heterogeneous Saline Formations: Long-Term Benefits for Geo-Sequestration of Greenhouse Gases.

[18] Powell S, Baker K (2009). Management Science. The Art of Modeling with Spreadsheets.

[19] Heddle G, Herzog H, Klett M (2003). The Economics of CO_2_ Storage.

[20] McCoy S (2008). The Economics of CO_2_ Transport by Pipeline and Storage in Saline Aquifers and Oil Reservoirs.

[21] Skovholt O (1993). CO_2_ transportation system. Energy Conv. Manage.

[22] Bakken BH, von Streng Velken I (2008). Linear Models for Optimization of Infrastructure for CO_2_ Capture and Storage. IEEE Trans. Energy Convers.

[23] Zhang ZX, Wang GX, Massarotto P, Rudolph V (2006). Optimization of pipeline transport for CO_2_ sequestration. Energy Conv. Manage.

[24] Mendelevitch R, Herold J, Oei P-Y, Tissen A (2010). CO_2_ Highways for Europe: Modelling a Carbon Capture, Transport and Storage Infrastructure for Europe.

[25] van den Broek M, Brederode E, Ramirez A, Kramers L, van der Kuip M, Wildenborg T, Turkenburg W, Faaij A (2010). Designing a cost-effective CO_2_ storage infrastructure using a GIS based linear optimization energy model. Environ. Modell. Softw.

[26] Middleton RS, Bielicki JM (2009). A scalable infrastructure model for carbon capture and storage: SimCCS. Energy Policy.

[27] Middleton RS, Bielicki JM (2009). A comprehensive carbon capture and storage infrastructure model. Energy Procedia.

[28] Keating GN, Middleton RS, Stauffer PH, Viswanathan HS, Letellier BC, Pasqualini D, Pawar RJ, Wolfsberg AV (2011). Mesoscale. Carbon sequestration site screening and CCS infrastructure analysis. Environ. Sci. Technol.

[29] Kuby M, Bielicki JM, Middleton RS (2011). Optimal spatial deployment of carbon dioxide capture and storage given a price on carbon dioxide. Int Reg Sci Rev.

[30] Lewis S (2010). 1Q Cost Report. Eng News Rec.

[31] Lewicki JL, Birkholzer J, Tsang C (2007). Natural and industrial analogues for leakage of CO_2_ from storage reservoirs: Identification of features, events, and processes and lessons learned. Environ. Geol.

[32] Cole WS (1942). Stratigraphic and Paleontologic Studies of Wells in Florida—No. 2. Florida Geological Survey.

[33] Chen CS (1965). The Regional Lithostratigraphic Analysis of Paleocene and Eocene Rocks of Florida. Florida Geological Survey.

[34] Babcock C (1969). Geology of the Upper Cretaceous Clastic Section Northern Peninsular Florida. Florida Geological Survey.

[35] Vernon RO (1970). The Beneficial Uses of Zones of High Transmissivity in the Florida Subsurface for Water Storage and Waste Disposal.

[36] Puri HS, Winston GO (1974). Geologic Framework of the High Transmissivity Zones in South Florida.

[37] Raymond DE, Copeland CW (1988). Alabama Stratigraphy.

[38] Rupert FR (1991). Geology of Gulf County, Florida.

[39] Yamamoto H, Zhang K, Karasaki K, Marui A, Uehara H, Nishikawa N (2009). Numerical investigation concerning the impact of CO_2_ geologic storage on regional groundwater flow. Int. J. Greenh. Gas Control.

[40] Okwen RT, Stewart MT, Cunningham J (2010). Analytical solution for estimating storage efficiency of geologic sequestration of CO_2_. Int. J. Greenh. Gas Control.

[41] Cormier G, Gunn EA (1999). Modelling and analysis for capacity expansion planning in warehousing. J. Oper. Res. Soc.

[42] Bayer MR, Kobelski B (2008). Underground injection control program: Proposed regulations for underground injection of carbon dioxide for geologic sequestration. Ground Water Monit. Remediat.

[43] Center for Climate Strategies (2008). Final Florida Greenhouse Gas Inventory and Reference Case Projections 1990–2025.

[44] Brennan ST, Burruss RC (2006). Specific storage volumes: A useful tool for CO_2_ storage capacity assessment. Nat. Resour. Res.

